# Chronic Inhibition of Dopamine β-Hydroxylase Facilitates Behavioral Responses to Cocaine in Mice

**DOI:** 10.1371/journal.pone.0050583

**Published:** 2012-11-27

**Authors:** Meriem Gaval-Cruz, Larry Cameron Liles, Paul Michael Iuvone, David Weinshenker

**Affiliations:** 1 Department of Human Genetics, Emory University School of Medicine, Atlanta, Georgia, United States of America; 2 Department of Ophthalmology, Emory University School of Medicine, Atlanta, Georgia, United States of America; University of Queensland, Australia

## Abstract

The anti-alcoholism medication, disulfiram (Antabuse), decreases cocaine use in humans regardless of concurrent alcohol consumption and facilitates cocaine sensitization in rats, but the functional targets are unknown. Disulfiram inhibits dopamine β-hydroxylase (DBH), the enzyme that converts dopamine (DA) to norepinephrine (NE) in noradrenergic neurons. The goal of this study was to test the effects of chronic genetic or pharmacological DBH inhibition on behavioral responses to cocaine using DBH knockout (*Dbh −/−*) mice, disulfiram, and the selective DBH inhibitor, nepicastat. Locomotor activity was measured in control (*Dbh +/−*) and *Dbh −/−* mice during a 5 day regimen of saline+saline, disulfiram+saline, nepicastat+saline, saline+cocaine, disulfiram+cocaine, or nepicastat+cocaine. After a 10 day withdrawal period, all groups were administered cocaine, and locomotor activity and stereotypy were measured. Drug-naïve *Dbh −/−* mice were hypersensitive to cocaine-induced locomotion and resembled cocaine-sensitized *Dbh +/−* mice. Chronic disulfiram administration facilitated cocaine-induced locomotion in some mice and induced stereotypy in others during the development of sensitization, while cocaine-induced stereotypy was evident in all nepicastat-treated mice. Cocaine-induced stereotypy was profoundly increased in the disulfiram+cocaine, nepicastat+cocaine, and nepicastat+saline groups upon cocaine challenge after withdrawal in *Dbh +/−* mice. Disulfiram or nepicastat treatment had no effect on behavioral responses to cocaine in *Dbh −/−* mice. These results demonstrate that chronic DBH inhibition facilitates behavioral responses to cocaine, although different methods of inhibition (genetic vs. non-selective inhibitor vs. selective inhibitor) enhance qualitatively different cocaine-induced behaviors.

## Introduction

The anti-alcoholism medication, disulfiram, has shown promise for reducing cocaine use in addicts in most, but not all studies, in a manner independent of alcohol intake, particularly at higher doses and in non-alcoholic subjects [1]–[13]. Acute disulfiram administration in rodents attenuates cocaine-induced locomotor activity, anxiety, and reinstatement of cocaine seeking [14]–[16], whereas chronic disulfiram exposure facilitates cocaine sensitization and cocaine-induced seizures [17], [18]. While promising, none of these studies were designed to identify the mechanisms underlying the ability of chronic disulfiram to alter cocaine-induced behaviors. Because the primary metabolite of disulfiram, N,N-diethyldithiocarbamate, is a copper chelator, it impairs the function of many copper-containing enzymes and produces side effects [2]. Identifying the functional targets of disulfiram, particularly those that underlie its chronic effects on cocaine responses, could lead to safer and more effective alternatives for the treatment of cocaine dependence.

Dopamine β-hydroxylase (DBH), the enzyme that converts DA into NE in noradrenergic neurons, requires copper and is inhibited by disulfiram, and disulfiram decreases NE and increases DA in both rodents and humans [2]. Disulfiram increases self-reported ratings of psychostimulant aversion, such as anxiety, nervousness, paranoia, craving, and dysphoria in humans [1], [7], [19]–[24], and addicts with polymorphisms in the *DBH* gene that confer low DBH activity report higher levels of cocaine-induced paranoia [25], [26]. Individuals with low DBH activity also appear to be particularly sensitive to disulfiram-induced psychosis [27]–[29]. In rodents, chronic disulfiram treatment or targeted disruption of the DBH gene each produce behavioral hypersensitivity to psychostimulants, including more pronounced cocaine aversion [18], [30], [31].

In this study, we used a combined genetic and pharmacological approach to determine whether disulfiram alters cocaine responses by inhibiting DBH. We predicted that (1) DBH knockout (*Dbh −/−*) mice would have altered responses to cocaine, (2) chronic disulfiram administration in control mice would produce a *Dbh −/−* like phenotype, (3) chronic disulfiram administration would have no consequences on cocaine responses in *Dbh −/−* mice, and (4) chronic administration of nepicastat, a drug that does not chelate copper but selectively inhibits DBH by binding the active site of the enzyme [32], [33], would mimic the behavioral profile of disulfiram.

## Materials and Methods

### Animals

Mice were separated by sex and housed 4–6 per cage at weaning, and adult (3- to 8-month-old) mice were used for all experiments. Food and water were available ad libitum throughout the course of the study, except during behavioral testing. No statistically significant sex differences were observed for cocaine responses (data not shown), and data from male and female mice were combined. *Dbh −/−* mice were generated as described [34] and maintained on a mixed C57Bl6/J and 129SvEv background. Homozygous *Dbh −/−* embryos die between E9.5–E12.5. To generate adult *Dbh −/−* mice, the embryonic lethal phenotype is rescued by spiking the drinking water of pregnant dams by adding the adrenergic receptor agonists phenylephrine and isoproterenol (20 µg/ml each) to the drinking water of pregnant dams from E9.5–E14.5, and adding the synthetic NE precursor L-3,4-dihydroxyphenylserine (L-DOPS; 2 mg/ml) from E14.5-parturition. After birth, no further pharmacological interventions are required for postnatal survival or development; thus, Dbh −/− mice lack norepinephrine from birth. However, even with these prenatal pharmacological interventions, Mendelian ratios of *Dbh −/−* mice are not obtained. To generate enough mice for this study, *Dbh −/−* males are crossed with *Dbh +/−* females, generating homozygous (*Dbh −/−*) and heterozygous (*Dbh +/−*) knockouts, but no "true" wild types (*Dbh +/+*). This has been the standard breeding scheme for all laboratories using *Dbh −/−* mice since their creation in 1995. Over 50 papers have been published using these mice, and most of them have used *Dbh +/−* mice as controls. The several exceptions that did generate and compare *Dbh +/+* and *Dbh +/−* mice found no behavioral, physiological, or neurochemical differences, justifying the use of *Dbh +/−* mice as controls [34]–[38]. Animals were treated in accordance with NIH policy, and experiments were approved by the Emory IACUC.

### Drugs

Cocaine-HCl was obtained from the NIDA Drug Supply Program and dissolved in sterile saline. Disulfiram (Sigma-Aldrich, St. Louis, MO) and nepicastat (Synosia Therapeutics, South San Francisco, CA) were sonicated in sterile saline and injected as a suspension. The typical therapeutic dose for disulfiram in the cocaine studies performed in humans is 250–500 mg/day [7], [22], which translates to ∼ 3–7 mg/kg for a 70 kg human, or ∼ 10-fold lower than we used in our study. Because of their higher metabolic rate, rodents require much larger doses of psychoactive drugs to produce behavioral and neurochemical effects compared to humans, and the 3–7 mg/kg dose has been shown to inhibit DBH in humans with a magnitude similar to the 100 mg/kg dose in rodents [29], [35], [39]–[41]. Thus, use of the 100 mg/kg dose in mice is a close functional match to therapeutic doses in humans. Furthermore, 100 mg/kg was the dose shown previously to facilitate cocaine sensitization in rodents [18]. We used the 50 mg/kg dose of nepicastat because it produces a reduction in brain NE levels similar to the 100 mg/kg dose of disulfiram (see Results). The phenylephrine and isoproterenol used for breeding *Dbh* mice were obtained from Sigma-Aldrich, and the L-DOPS was a gift from Dainippon Sumitomo Pharma (Osaka, Japan).

### Quantification of Norepinephrine Levels

Mice were injected with saline (10 ml/kg, i.p.) or nepicastat (50 mg/kg, i.p.) 3 times, each injection two hours apart. Two hours after the last injection, mice were euthanized by CO_2_ asphyxiation and brains were removed and dissected on ice, and frozen. The frontal cortex was isolated by removing the olfactory bulb and making a cut 1 mm posterior to the frontal pole. NE levels were determined using HPLC followed by coulometric detection. NE concentrations were normalized to wet tissue weight for each sample.

Analytical samples of saline- and nepicastat-treated mice were prepared by adding 70 µL of ice-cold 0.1 N perchloric acid and 0.04% sodium metabisulfite to the tissue, and then sonicating until completely homogenized. Samples were centrifuged at 15 rpm x 1000 for 10 minutes at 4°C. This supernatant was injected at a constant flow rate of 1 mL/min onto an Ultrasphere ODS 250 × 4.6 mm column, 5 µm (Beckman Coulter, Fullerton, CA, USA) with mobile phase (0.1 mM EDTA; 0.35 mM sodium octyl sulfate; 0.6% phosphoric acid; 5% acetonitrile (pH 2.7)). A coulometric electrochemical array detector (ESA Biosciences; guard cell set at 600 mV and analytical cell at 300 mV) was used to visualize the peaks. The retention time, height, and area of NE peaks were compared with reference standard solutions (Sigma-Aldrich, St. Louis, MO) and quantified by ChemStation chromatography software (Agilent Technologies).

### Cocaine Administration Paradigm

**Figure 1:**
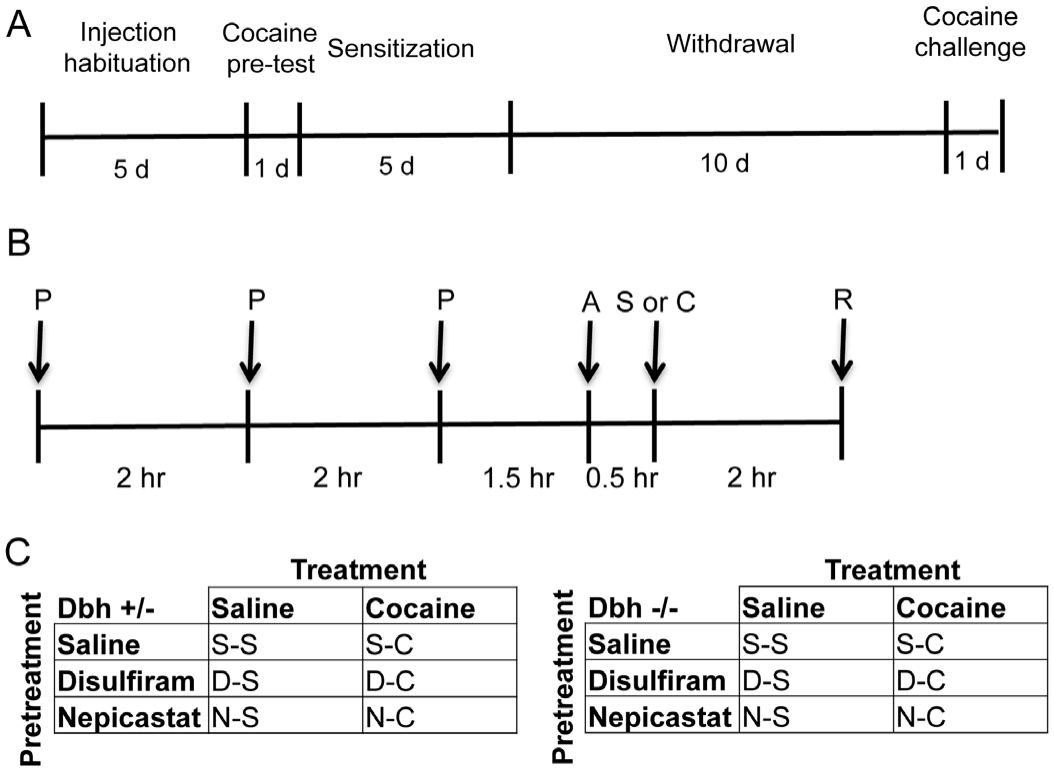
Drug administration paradigm. (A) Timeline for behavioral testing. (B) Timeline for injections and activity recording on each of the 5 cocaine sensitization days. P, pretreatment (saline, disulfiram, or nepicastat); A, mice placed in activity chambers; S, saline injection; C, cocaine injection; R, mice returned to their home cage. (C) Pretreatment and treatment groups for each genotype.

The behavioral testing timeline, similar to the one used previously that revealed facilitated cocaine sensitization following chronic disulfiram administration [18], is shown in Fig. 1A. Adult *Dbh* +/− and *Dbh* −/− mice were injected in their home cage with saline (10 ml/kg, i.p.) 4 times per day, each injection 2 hours apart, for 5 days before the pretest day to habituate them to the total volume of the injections. On the sixth day, mice were placed in locomotor activity recording chambers and allowed to habituate for 30 minutes before receiving a single injection of cocaine (15 mg/kg, i.p.), and their locomotor activity was recorded for an additional 2 hours (“Pretest” day). Ambulations (consecutive beam breaks) were recorded in transparent Plexiglas cages placed into a rack with 7 infrared photobeams, each spaced 5 cm apart (San Diego Instruments Inc., La Jolla, CA).

Mice were then assigned to treatment groups with similar within-genotype cocaine-induced locomotor activity scores from Pretest day. Cocaine sensitization took place on the following 5 consecutive days (see Fig. 1B for daily sensitization timeline). For the DBH inhibitor treatments, we used a published design for which disulfiram altered brain NE levels and cocaine-induced locomotor activity [14]. Mice were pretreated with saline, disulfiram (100 mg/kg, i.p.), or nepicastat (50 mg/kg, i.p.), 3 times per day, each injection spaced 2 hours apart. Ninety minutes following the last pretreatment, mice were placed in activity chambers, injected with saline or cocaine (15 mg/kg, i.p.) 30 minutes later, and locomotor activity was recorded for an additional 2 hours. Thus, there were 12 groups of mice total: *Dbh +/−* saline+saline (male, n = 3; female, n = 4), *Dbh +/−* disulfiram+saline (male, n = 2; female, n = 6), *Dbh +/−* nepicastat+saline (male, n = 3; female, n = 4), *Dbh +/−* saline+cocaine (male, n = 4; female, n = 5), *Dbh +/−* disulfiram+cocaine (male, n = 6; female, n = 7), *Dbh +/−* nepicastat+cocaine (male, n = 4; female, n = 3), *Dbh −/−* saline+saline (male, n = 4; female, n = 3), *Dbh −/−* disulfiram+saline (male, n = 3; female, n = 4), *Dbh −/−* nepicastat+saline (male, n = 4; female, n = 2), *Dbh −/−* saline+cocaine (male, n = 4; female, n = 4), *Dbh −/−* disulfiram+cocaine (male, n = 3; female, n = 4), and *Dbh −/−* nepicastat+cocaine (male, n = 2; female, n = 2) (Fig. 1C).

Following the last injection on the fifth day of treatment, animals were placed back in their home cage and left undisturbed for a 10 day withdrawal period. The next day, all mice were placed in the activity chambers for 30 minutes, then given a challenge injection of cocaine (15 mg/kg, i.p.). Locomotor activity was recorded for an additional 2 hours, and mice were scored for the appearance of stereotypic behaviors by a trained observer blind to experimental conditions. Horizontal locomotion and rearing were considered normal exploratory behaviors, while repetitive head-bobbing, sniffing, circling, and nail biting were considered as stereotypy, as described [31]. Behavior was quantified for 5 min, ∼15 min following cocaine administration. Circling was the predominant stereotypyical behavior observed, followed by repetitive head bobbing and sniffing. In general, there was an “all or none” response; mice either spent greater than 50% of the observation period engaged in stereotypical behaviors, and were classified in the “stereotypy” group, or they spent virtually none of the observation period engaged in these behaviors, and were classified in the “no stereotypy” group.

### Statistics

Depending on the experiment, data were analyzed by Chi-square, One-way ANOVA, repeated measures One-way ANOVA, or repeated measures Two-way ANOVA, followed by posthoc tests, where appropriate. Prism 6.0 for Macintosh was used for all statistical analysis.

## Results

### Dbh −/− Mice are Hypersensitive to Cocaine-induced Locomotion

**Figure 2:**
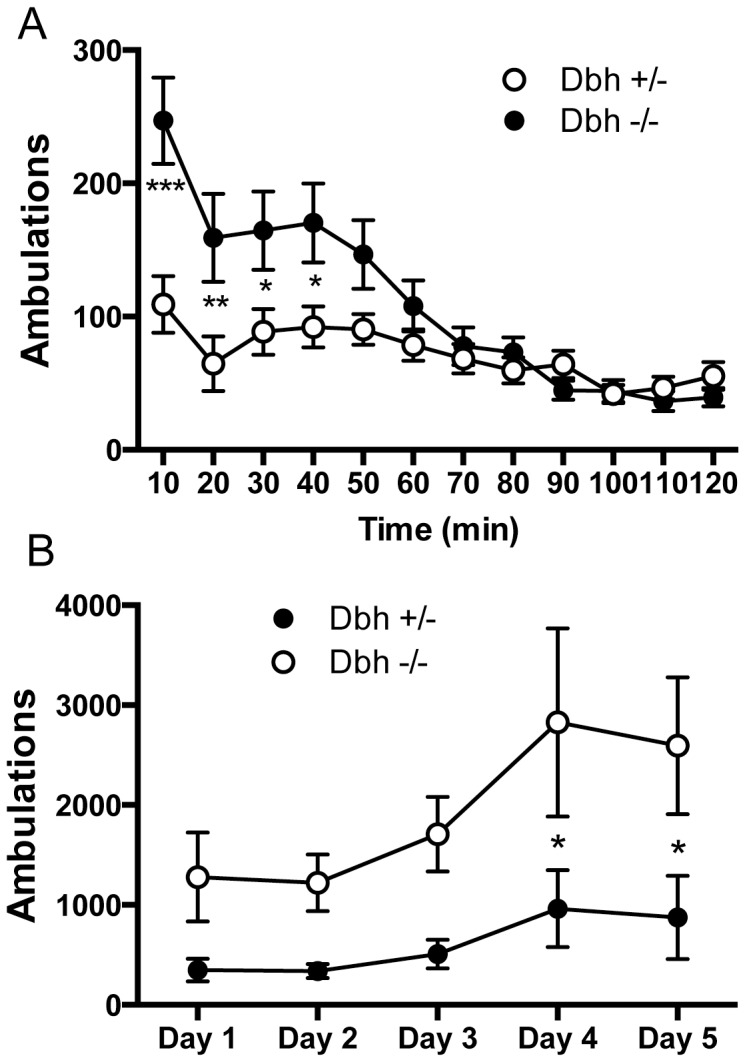
*Dbh −/−* mice are hypersensitive to cocaine-induced locomotion. (A) Drug-naïve *Dbh +/−* (n = 9) and *Dbh −/−* mice (n = 8) were placed in automated locomotor activity chambers, injected with cocaine (15 mg/kg, i.p.) 30 minutes later, and locomotor activity was recorded for 2 hours. Shown are mean ± SEM ambulations (consecutive beam breaks). *** *p*<0.0001, ** *p*<0.01, * *p*<0.05 compared with *Dbh −/−* mice at that time point. (B) On each of the next 5 days, mice were administered saline (3 injections of 10 ml/kg, each injection spaced 2 hours apart). Ninety minutes after the last saline injection, mice were placed in automated locomotor activity chambers, injected with cocaine (15 mg/kg, i.p.) 30 minutes later, and locomotor activity was recorded for 2 hours. Shown are mean ± SEM ambulations (consecutive beam breaks) for the 2 hours following cocaine administration. * p<0.05 between genotypes for that day.

As we reported before for other doses of cocaine (5, 10, and 15 mg/kg) [30], drug-naïve *Dbh −/−* mice were hypersensitive to the 15 mg/kg dose of cocaine we used for our sensitization experiments compared to *Dbh +/−* mice on pretest day (Fig. 2A). Repeated measures 2-way ANOVA revealed a main effect of genotype (F_1,792_ = 4.79, *P*<0.05), time (F_11,792_ = 23.46, *P*<0.0001), and a genotype x time interaction (F_11,792_ = 7.93, *P*<0.0001) (Fig. 2). Posthoc tests showed that locomotor activity was significantly greater in *Dbh −/−* mice at the 10-, 20-, 30-, and 40-minutes time points following cocaine administration. This hypersensitivity persisted during the development of sensitization over 5 days of cocaine administration (Fig. 2B). Repeated measures 2-way ANOVA revealed a main effect of genotype (F_1,15_ = 6.73, *P*<0.05) and time (F4,60 = 6.38, *P*<0.001). Although posthoc tests showed that locomotor activity was significantly greater in *Dbh −/−* mice only on days 4 and 5, the magnitude of the difference (approximately 3-fold) appeared similar on all days (Fig. 2B).

### Nepicastat Reduces Brain NE content in Dbh +/− Mice

We reported before that disulfiram (3 x 100 mg/kg, i.p, each injection 2 hr apart, brain samples taken 2 hr after the last injection) reduces tissue NE levels in the frontal cortex of *Dbh +/−* mice by ∼50% [35]. A similar dosing regimen with nepicastat (50 mg/kg, i.p.) produced a slightly greater (∼75%) reduction in cortical NE levels (vehicle, 0.18±0.01 ng/mg tissue, *n* = 12; nepicastat, 0.04±0.01 ng/mg tissue, *n* = 13, *P*<0.0001). Although we did not measure dopamine or serotonin levels in this study, we have previously published data showing that disulfiram and nepicastat decrease norepinephrine and increase dopamine, and that disulfiram has no effect on serotonin levels (Bourdelat-Parks et al., 2005; Schroeder et al., 2010).

### Effects of Chronic Disulfiram and Nepicastat Administration on the Development of Cocaine Sensitization in Dbh +/− Mice

**Figure 3:**
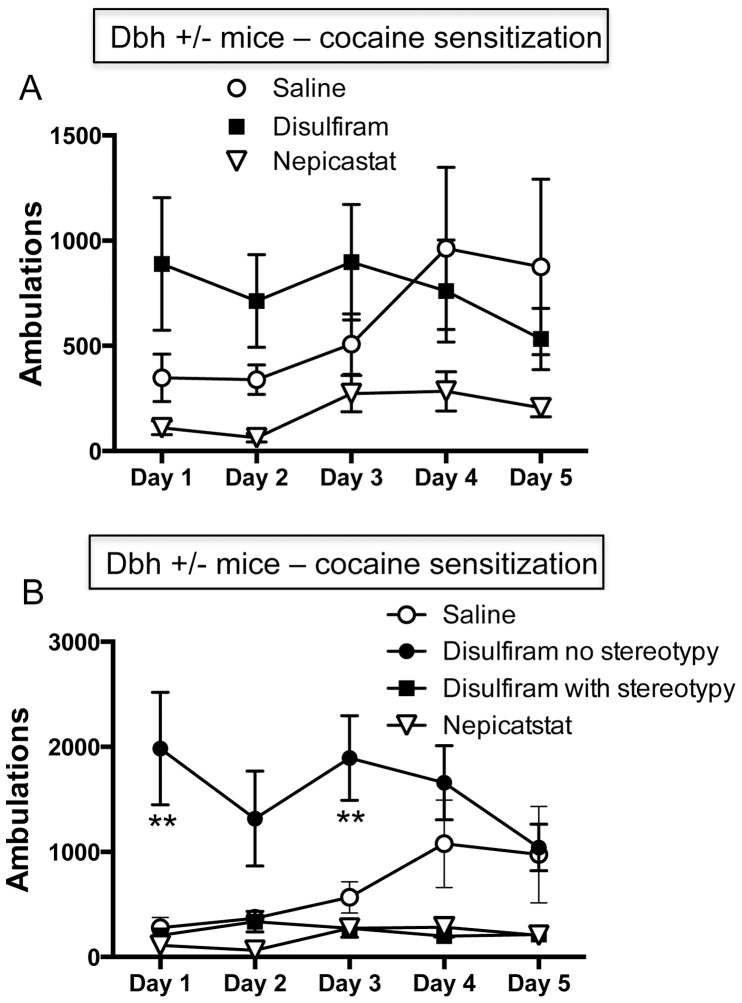
Effects of chronic disulfiram or nepicastat administration on cocaine-induced locomotor activity in *Dbh +/−* mice during the development of sensitization. Each day for 5 consecutive days, *Dbh +/−* mice were administered saline (n = 9), disulfiram (3 injections of 100 mg/kg, i.p., each injection spaced 2 hours apart; n = 13), or nepicastat (3 injections of 50 mg/kg, i.p., each injection spaced 2 hours apart; n = 7). Ninety minutes after the last pretreatment, mice were placed in automated locomotor activity chambers, injected with cocaine (15 mg/kg, i.p.) 30 minutes later, and locomotor activity was recorded for 2 hours. (A) shows data with all disulfiram-treated mice in a single group. (B) shows data with disulfiram-treated mice divided into “no stereotypy” and “with stereotypy” groups. * p<0.01 compared to saline-treated group.

Fig. 3A shows the locomotor activity of *Dbh +/−* mice over the 5 day sensitization regimen in the mice receiving daily cocaine injections (the saline+cocaine, disulfiram+cocaine, and nepicastat+cocaine groups). Compared to the saline-cocaine group, chronic treatment of *Dbh +/−* mice with disulfiram tended to increase cocaine-induced locomotor activity during the first 3 days of the sensitization regimen. For example, the locomotor activity of the disulfiram+cocaine group on day 1 was comparable to that of the fully sensitized saline-cocaine group on day 5. By contrast, nepicastat tended to suppress cocaine-induced locomotor activity over the 5 day sensitization period. However, none of these trends reached statistical significance for pretreatment (F(2,26) = 2.09, *p* = 0.14), time (F(4,104) = 1.32, *p* = 0.27), or a pretreatment x time interaction (F(8,104) = 1.61, *p* = 0.13).

Behavioral responses to cocaine are dose-dependent; low to moderate doses increase locomotor activity, while higher doses or repeated cocaine administration produce stereotypies at the expense of locomotor activity [42]–[44]. During visual inspection of the mice following cocaine administration, we noticed that behavioral sensitization to cocaine manifested in these two distinct ways; some mice showed increased cocaine-induced locomotor activity over the course of the experiment, while in other mice, cocaine-induced locomotor activity was replaced by intense stereotypy. Stereotypy was defined as non-goal-directed, repetitive behaviors, such as circling, head-bobbing, nail biting or repetitive sniffing (see Materials and Methods). All but one saline-pretreated *Dbh +/−* mice displayed increased locomotor activity, rather than stereotypy, in response to cocaine over time. By contrast, some disulfiram-pretreated mice showed hypersensitivity to cocaine-induced locomotion that was similar in magnitude to that seen in saline-pretreated mice after 5 days of cocaine, while the rest of the disulfiram-treated mice rapidly developed cocaine-induced stereotypy at the expense of increased locomotor activity. All of the nepicastat-pretreated mice developed stereotypy in response to cocaine, instead of increased locomotor activity. These observations led us to reanalyze the data from the disulfiram-treated mice in two groups - those that developed stereotypy and those that did not – and clear differences emerged (Fig. 3B). We also quantified stereotypy following cocaine challenge after a 10 day withdrawal period (see below). The disulfiram-treated mice that did not display stereotypy had much greater cocaine-induced locomotor activity than the saline-pretreated mice, while cocaine-induced locomotor activity was very low in those disulfiram-treated mice that developed stereotypy. Repeated measures 2-way ANOVA analysis revealed a main effect of pretreatment (F(3,24) = 15.13, *p*<0.0001) and a pretreatment x time interaction (F(12,96) = 2.13, *p*<0.05). Posthoc analysis showed that the disulfiram-pretreated mice not engaged in stereotypy had significantly increased cocaine-induced locomotor activity versus the saline-pretreated mice on days 1 and 3.

Locomotor activity in the animals receiving chronic saline injections (the saline+saline, disulfiram+saline, and nepicastat+saline groups) was very low and unaffected by DBH inhibitor treatment (data not shown).

### Effects of Chronic Disulfiram and Nepicastat Administration on the Expression of Cocaine Sensitization in Dbh +/− Mice

**Figure 4:**
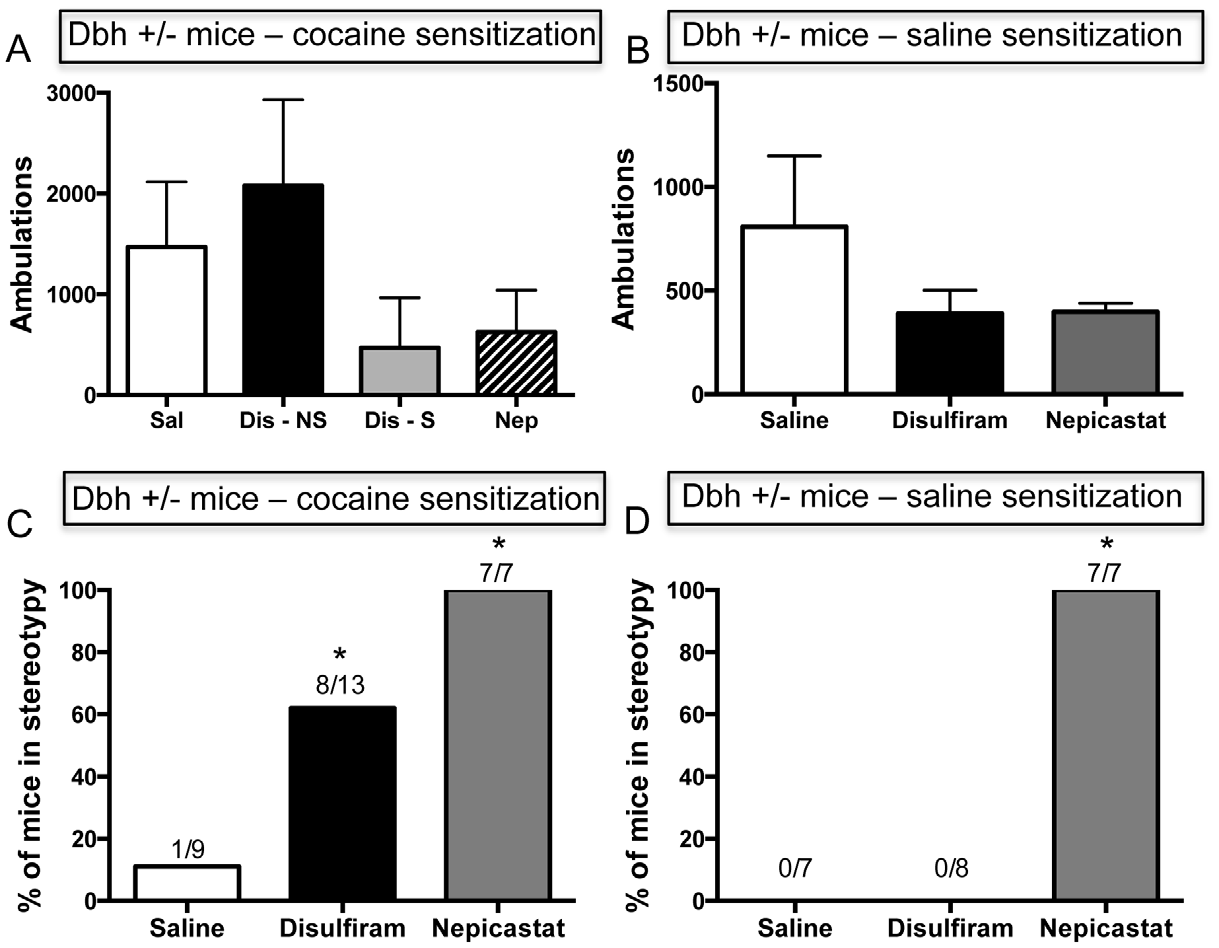
Effects of chronic disulfiram or nepicastat administration on cocaine-induced locomotor activity and stereotypy in *Dbh +/−* mice during the expression of sensitization. Ten days following the 5 day sensitization paradigm (see Fig. 3 legend), all mice were placed in the activity chambers for 30 min, given an injection of cocaine (15 mg/kg, i.p.), and locomotor activity was recorded for 2 hr and stereotypy was scored. (A) Mean ± SEM ambulations for mice in the groups that received cocaine injections during the 5 day sensitization period (saline+cocaine (“Sal”), n = 9; disulfiram+cocaine that did not display stereotypy (“Dis - NS”), n = 5; disulfiram+cocaine that displayed stereotypy (“Dis - S”), n = 8; nepicastat+cocaine (“Nep”), n = 7). (B) Mean ± SEM ambulations for mice in the groups that received saline injections during the 5 day sensitization period (saline+saline, n = 7; disulfiram+saline, n = 8; nepicastat+saline, n = 7). (C) Percentage of mice in the groups that received cocaine injections during the 5 day sensitization period (saline+cocaine, disulfiram+cocaine, nepicastat+cocaine) that primarily engaged in stereotypy. (D) Percentage of mice in the groups that received saline injections during the 5 day sensitization period (saline+saline, disulfiram+saline, nepicastat+saline) that primarily engaged in stereotypy following cocaine challenge. * p<0.05 compared with the saline control for that group (saline+cocaine for panel C, saline+saline for panel D).

Cocaine-induced locomotor activity in *Dbh +/−* mice on challenge day following a 10 day withdrawal period is shown in Fig. 4A and 4B. Disulfiram tended to increase the expression of cocaine-induced locomotor activity in those mice that did not display stereotypy (the disulfiram+cocaine “no stereotypy” group, while both disulfiram and nepicastat tended to decrease the expression of cocaine-induced locomotor sensitization in the animals that were primarily engaged in stereotypy following cocaine injections during the 5 day sensitization period (the disulfiram+cocaine “with stereotypy” and nepicastat+cocaine groups compared to the saline+cocaine group) (Fig. 4A), but the differences did not quite reach statistical significance (one-way ANOVA; F(3,24) = 2.08, p = 0.13). Less pronounced, non-significant reductions on cocaine-induced locomotor activity on challenge day were evident in the animals that received saline injections during the 5 day sensitization period (the disulfiram+saline and nepicastat+saline groups compared to the saline+saline group) (Fig. 4B).

Cocaine-induced stereotypy in *Dbh +/−* mice on challenge day, following a 10 day withdrawal period, is shown in Fig. 4C and 4D. Stereotypy was defined as non-goal-directed, repetitive behaviors, such as circling, head-bobbing, nail biting or repetitive sniffing (see Materials and Methods). The percentage of mice that developed cocaine-induced stereotypy was significantly higher in the disulfiram+cocaine (62%) and nepicastat+cocaine (100%) groups compared to the saline+cocaine group (11%) (Fig. 4C) (Chi-square = 12.97, *P*<0.01). These mice that displayed stereotypy on challenge day were the same ones that were engaged primarily in stereotypy following cocaine administration during the 5 day sensitization period. In addition, all of the mice in the nepicastat+saline group engaged in stereotypy following cocaine administration on challenge day, whereas none of the saline+saline or disulfiram+saline mice did (Fig. 4D) (Chi-square = 22, *P*<0.0001). All mice in the “stereotypy” category spent most (>90%) of the observation time following cocaine administration engaged in stereotypic behaviors.

### The Development and Expression of Cocaine Sensitization in Dbh −/− Mice are Unaffected by Disulfiram or Nepicastat

**Figure 5:**
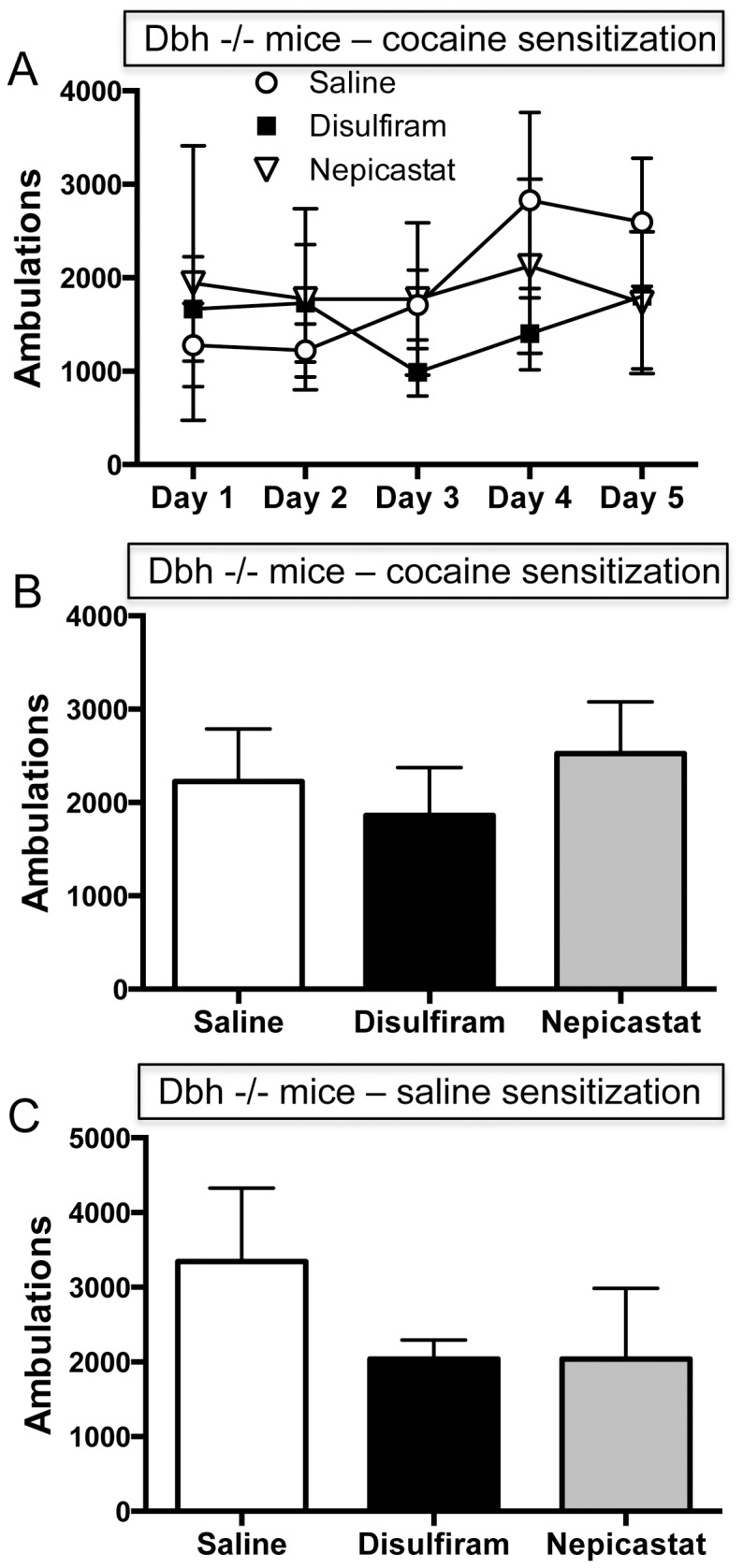
Effects of chronic disulfiram or nepicastat administration on cocaine-induced locomotor activity in *Dbh −/−* mice during the development and expression of sensitization. *Dbh −/−* mice were put through the 5 day sensitization paradigm followed by cocaine challenge after 10 days of withdrawal paradigm (see Fig. 3 and Fig. 4 legends). (A) Mean ± SEM ambulations for the 2 hours following cocaine administration during the 5 day sensitization period sensitization in the groups that received cocaine injections (saline+cocaine, n = 8; disulfiram+cocaine, n = 7; nepicastat+cocaine, n = 4). (B) Mean ± SEM ambulations for the 2 hours following cocaine challenge after 10 days of withdrawal in the groups that received cocaine injections during the 5 day sensitization period (saline+cocaine; disulfiram+cocaine; nepicastat+cocaine). (C) Mean ± SEM ambulations for the 2 hours following cocaine challenge after 10 days of withdrawal in the groups that received saline injections during the 5 day sensitization period (saline+saline, n = 7; disulfiram+saline, n = 7; nepicastat+saline, n = 6).

If disulfiram is facilitating cocaine sensitization via DBH inhibition, then neither disulfiram nor nepicastat should alter behavior in mice lacking DBH, and we found that this was indeed the case. The DBH inhibitors did not significantly affect locomotor activity in *Dbh −/−* mice during the 5 day sensitization period in the groups receiving cocaine injections (saline+cocaine, disulfiram+cocaine, and nepicastat+cocaine) (Fig. 5A). Locomotor activity in the *Dbh −/−* groups receiving saline injections (saline+saline, disulfiram+saline, and nepicastat+saline) was very low, and was likewise unaffected by DBH inhibitors (data not shown). Disulfiram and nepicastat also did not alter cocaine induced locomotor activity on challenge day following a 10 day withdrawal period in the *Dbh −/−* groups that received cocaine or saline during the 5 day sensitization period (Fig. 5B and 5C, respectively). Most importantly, the ability of disulfiram and nepicastat to increase the incidence of cocaine-induced stereotypy was abolished in *Dbh −/−* mice; no stereotypy was observed in *Dbh −/−* mice during the 5 day sensitization period or on challenge day in any treatment group.

## Discussion

The purpose of this study was to determine the effects of chronic DBH inhibition on cocaine responses, and whether the disulfiram-induced facilitation of cocaine sensitization, reported previously in rats [18], was due to disulfiram’s ability to inhibit DBH. Although many studies have speculated that disulfiram alters cocaine responses via DBH inhibition, they all lacked a DBH-deficient control group, a selective DBH inhibitor, chronic disulfiram administration, and/or assessment of brain NE levels, and thus were not designed to test the contribution of DBH. To get around these limitations, we employed a combination of chronic disulfiram administration, mice lacking DBH completely (*Dbh −/−* mice), a selective DBH inhibitor (nepicastat), and brain neurochemistry.

### Chronic DBH Deficiency Enhances Behavioral Responses to Cocaine

The results presented here confirm and extend our previous finding that *Dbh −/−* mice are hypersensitive to cocaine-induced locomotion [30]. First, we showed that drug-naïve *Dbh −/−* mice are hypersensitive to a dose of cocaine (15 mg/kg) that had not previously been tested. Second, we found that the increased cocaine-induced locomotion in *Dbh −/−* mice persisted over a 5 day sensitization regimen. Finally, *Dbh −/−* mice appear to have a “pre-sensitized”-like phenotype; cocaine-induced locomotion in drug-naïve *Dbh −/−* mice was comparable, if not greater, to that of fully sensitized *Dbh +/−* mice after 5 days of cocaine.

Daily pretreatment of *Dbh +/−* mice with DBH inhibitors had a complex effect on cocaine-induced locomotor behavior. Disulfiram increased cocaine-induced locomotion in some mice, and produced intense stereotypy in response at the expense of horizontal locomotion to cocaine in others, while nepicastat pretreatment produced stereotypy in all mice receiving cocaine during the 5 day sensitization paradigm. This pattern of behavior (decreased locomotion and increased stereotypy) are typically seen with very high doses of psychostimulants and/or chronic drug exposure [42], indicating that the stereotypy we observed likely represents enhanced behavioral responding to cocaine. Although disulfiram and nepicastat each produced similar behavioral hypersensitivity to cocaine, there were qualitative differences. Stereotypy was observed in all of the mice in the nepicastat+cocaine group, compared with ∼60% of the mice in the disulfiram+cocaine group. Furthermore, chronic nepicastat that was not paired with cocaine administration during the 5 day sensitization period (the nepicastat+saline group) was sufficient to produce stereotypy in response to cocaine after the 10 day withdrawal period on challenge day, while chronic disulfiram administration that was not paired with cocaine (the disulfiram+saline group) was not. The greater reduction in brain NE levels produced by nepicastat (∼75%) versus disulfiram (∼50%) could account for this difference. Alternatively, interaction of disulfiram with targets other than DBH may partially interfere with its ability to facilitate cocaine-induced stereotypy. *Dbh −/−* mice were hypersensitive to cocaine-induced locomotion, but stereotypy was not seen in this study or in previous experiments using even higher doses of cocaine [17], [30]. The reason for this finding is unclear, as *Dbh −/−* mice are capable of stereotypic behaviors, and in fact are more sensitive to amphetamine-induced stereotypy than *Dbh +/−* mice [31]. We speculate that this qualitative difference in behavioral cocaine hypersensitivity is due to compensatory effects that result from a lifetime of complete DBH inhibition (*Dbh −/−*), compared with the partial, five-day DBH inhibition in mice with otherwise normal catecholamine content. Combined, all of these results suggest that disulfiram facilitates cocaine sensitization by inhibiting DBH; the ability of disulfiram to enhance cocaine-induced stereotypy is shared by a selective DBH inhibitor and abolished in the absence of DBH.

### Potential Mechanisms Underlying Cocaine Hypersensitivity following DBH Inhibition

Disulfiram inhibits two enzymes involved in cocaine metabolism, cholinesterase and carboxylesterase [45]–[47], and increases peak serum cocaine levels in humans [19]–[22]. This means that one explanation for our results could be that genetic or pharmacological DBH inhibition impairs cocaine metabolism, and thus the mice may simply be experiencing higher concentrations of cocaine. However, we showed before that neither DBH knockout nor disulfiram affected peak serum cocaine levels in mice [3]. Furthermore, preliminary results indicate that nepicastat has no effect on cocaine metabolism in humans [48].

NE supplies excitatory drive onto midbrain DA neurons, and blockade of adrenergic receptors or NE synthesis impairs DA neuron firing and DA release [49]. Thus, while genetic or pharmacological inhibition of DBH increases tissue DA levels in the brain, basal and stimulant-induced increases in extracellular DA are reduced, which can explain the attenuation of behavioral responses to psychostimulants following acute DBH inhibition. However, in response to chronically low levels of extracellular DA, there are compensatory increases in postsynaptic DA receptor signaling, leading to cocaine hypersensitivity [2], [30], [49].

There is a recent report that acute disulfiram administration actually increases basal and cocaine-induced extracellular DA levels specifically in the PFC [50], which is inconsistent with previous DBH knockout data, DBH inhibitor data, and reduced cocaine-induced DA release in the PFC of cocaine-sensitized animals [30], [51], [52]. A lower dose of disulfiram (50 mg/kg) was used in that study, and recent evidence suggests that low doses of disulfiram increase, rather than decrease, cocaine use in humans [3]. It is also possible that the effects of DBH inhibitors on cocaine sensitization involve other neurotransmitter systems. For example, NE appears to modulate glutamate transmission within the mesocorticolimbic system, which is critical for cocaine-induced behavioral sensitization [52]–[54]. The effects of disulfiram on cocaine-induced neurotransmitter overflow and behavioral responses to cocaine warrant further investigation.

### Clinical Implications

Because disulfiram appears to facilitate cocaine sensitization, at least in part, via DBH inhibition, an important question is whether this mechanism contributes to disulfiram’s ability to reduce cocaine use in addicts, and if so, how. Cocaine sensitization in animals may represent an increase of the incentive motivational effects of the drug [55], suggesting that decreasing sensitization could treat addiction. However, to our knowledge, no medications have been identified that inhibit cocaine sensitization in animals and reliably reduce cocaine use in addicts. By contrast, disulfiram is clinically effective (albeit modestly), yet enhances cocaine sensitization. How can we reconcile these ideas and data? It has been known for a long time that humans also sensitize to the aversive properties of psychostimulants, such as stereotypy and paranoia [55]. Disulfiram deters alcohol consumption by inhibiting aldehyde dehydrogenase and creating an aversive response to alcohol; similarly, disulfiram may be “sensitizing” the aversive effects of cocaine via DBH inhibition, thereby reducing its use. Disulfiram is reported to increase psychostimulant-induced anxiety, nervousness, paranoia, and “bad drug effects” in humans [19]–[24]. Interestingly, *Dbh −/−* mice develop a conditioned place preference to cocaine at low doses (5 mg/kg) that do not support a place preference in control animals but develop a conditioned place aversion to cocaine at higher doses (20 mg/kg) that produce a place preference in control animals [30], and a recent study suggests that low disulfiram doses increase, while high disulfiram doses decrease the rewarding effects of cocaine in humans (C. Haile, personal communication). Humans with genetically low DBH activity report elevated levels of cocaine-induced paranoia [25], [26], and incidents of disulfiram-induced psychosis have been reported specifically in individuals with low intrinsic DBH activity [27], [29]. If cocaine aversion due to the inhibition of DBH by disulfiram accounted for its clinical efficacy, DBH alleles that conferred low activity might be underrepresented in addict populations [2], [56]. There has been only one large published study investigating this possibility, and no effect of DBH genotype was found [57]. It will be important to pursue further studies of this kind in other cocaine-dependent cohorts. NE transmission is also critical for relapse-like behavior triggered by drug re-exposure, cues, and stress [58]–[61], and we have found that acute disulfiram and/or nepicastat can attenuate cocaine-, cue-, yohimbine-, and footshock-induced reinstatement of cocaine seeking in rats [16] (our unpublished data). We propose that disulfiram reduces cocaine use initially by increasing the aversive properties of cocaine, then promotes abstinence by interfering with the ability of environmental triggers to precipitate drug seeking and relapse. Finally, because the clinical use of disulfiram as a pharmacotherapy for cocaine dependence is limited by the drug’s lack of specificity, its side effects and toxicity, other, more selective DBH inhibitors, such as nepicastat, need to be developed and tested in cocaine-dependent cohorts.
